# Intermittent theta burst stimulation enhances upper limb motor function in patients with chronic stroke: a pilot randomized controlled trial

**DOI:** 10.1186/s12883-019-1302-x

**Published:** 2019-04-25

**Authors:** Yu-Jen Chen, Ying-Zu Huang, Chung-Yao Chen, Chia-Ling Chen, Hsieh-Ching Chen, Ching-Yi Wu, Keh-Chung Lin, Tzu-ling Chang

**Affiliations:** 1 Department of Physical Medicine and Rehabilitation, Chang Gung Memorial Hospital, Linkou, 5, Fushing Street, Kuei-Shan District, Taoyuan City, 33305 Taiwan; 2 Neuroscience Research Center and Department of Neurology, Chang Gung Memorial Hospital, Linkou, Taiwan; 3 Institute of Cognitive Neuroscience, National Central University, Taoyuan, Taiwan; 4 Medical School, College of Medicine, Chang Gung University, Taoyuan, Taiwan; 5 Department of Physical Medicine and Rehabilitation, Chang Gung Memorial Hospital, Keelung, Taiwan; 6 Graduate Institute of Early Intervention, Chang Gung University, Taoyuan, Taiwan; 7 Department of Industrial and Management, National Taipei University of Technology, Taipei, Taiwan; 8 Department of Occupational Therapy, College of Medicine, Chang Gung University, Taoyuan, Taiwan; 9 School of Occupational Therapy, College of Medicine, National Taiwan University, Taipei, Taiwan; 10 Division of Occupational Therapy, Department of Physical Medicine and Rehabilitation, National Taiwan University Hospital, Taipei, Taiwan

**Keywords:** Transcranial magnetic stimulation (TMS), Theta burst stimulation (TBS), Stroke, Motor function, Rehabilitation

## Abstract

**Background:**

Intermittent theta burst stimulation (iTBS) is a form of repetitive transcranial stimulation that has been used to enhance upper limb (UL) motor recovery. However, only limited studies have examined its efficacy in patients with chronic stroke and therefore it remains controversial.

**Methods:**

This was a randomized controlled trial that enrolled patients from a rehabilitation department. Twenty-two patients with first-ever chronic and unilateral cerebral stroke, aged 30–70 years, were randomly assigned to the iTBS or control group. All patients received 1 session per day for 10 days of either iTBS or sham stimulation over the ipsilesional primary motor cortex in addition to conventional neurorehabilitation. Outcome measures were assessed before and immediately after the intervention period: Modified Ashworth Scale (MAS), Fugl-Meyer Assessment Upper Extremity (FMA-UE), Action Research Arm Test (ARAT), Box and Block test (BBT), and Motor Activity Log (MAL). Analysis of covariance was adopted to compare the treatment effects between groups.

**Results:**

The iTBS group had greater improvement in the MAS and FMA than the control group (*η*^*2*^ = 0.151–0.233; *p* < 0.05), as well as in the ARAT and BBT (*η*^*2*^ = 0.161–0.460; *p* < 0.05) with large effect size. Both groups showed an improvement in the BBT, and there were no significant between-group differences in MAL changes.

**Conclusions:**

The iTBS induced greater gains in spasticity decrease and UL function improvement, especially in fine motor function, than sham TBS. This is a promising finding because patients with chronic stroke have a relatively low potential for fine motor function recovery. Overall, iTBS may be a beneficial adjunct therapy to neurorehabilitation for enhancing UL function. Further larger-scale study is warranted to confirm the findings and its long-term effect.

**Trial registration:**

This trial was registered under ClinicalTrials.gov ID No. NCT01947413 on September 20, 2013.

## Background

Stroke is the leading cause of long-term disability globally [1]. It is estimated that 55–75% of post-stroke patients experience upper limb (UL) functional limitation [2]. This UL functional impairment results in restrictions on functional tasks and daily activities [3], and may, therefore, lead to decreased health-related quality of life [4]. A reported 50–60% of patients experience variable degrees of motor function limitation following stroke, even when undergoing traditional rehabilitation programs [5]. Repetitive transcranial magnetic stimulation (rTMS) is a promising emerging non-invasive brain stimulation technique for facilitating functional recovery by modulating neuroplastic processes, together with post-stroke motor network activity and connectivity [6]. However, one meta-analysis [7] stated that the current literature is not sufficient to show the superiority of rTMS combined with UL training over UL training alone. Intermittent TBS (iTBS), however, may hold more promise; thus, we investigated its efficacy in the neurorehabilitation of UL dysfunction.

Theta burst stimulation (TBS) is a novel form of rTMS composed of short and low-intensity bursts of rTMS at 50 Hz [8]. Compared to conventional rTMS protocols, TBS yields consistent and long-lasting effects on motor-evoked potentials (MEPs) after a shorter stimulation duration [8]. In general, iTBS intermittently produces short TBS trains to facilitate cortical excitability of primary motor cortex (M1), while continuous TBS (cTBS) represents a longer and continuous TBS train that suppresses M1 cortical excitability. Notably, loci other than M1 may not induce similar effects [9, 10]. Recent studies have shown controversial effects of TBS on UL function in patients with chronic stroke [11–16]. Based on the interhemispheric competition model, transcallosal inhibitory signals from the contralesional hemisphere hamper recovery of motor function post stroke [17], while cortical hyperexcitability in the contralesional hemisphere decreases with time after stroke [18] and may thus limit the effect of cTBS during the chronic stage. On the contrary, motor recovery has been associated with activation of the ipsilesional motor cortex in the chronic stroke stage [19], thus supporting the stimulation strategy of facilitating the ipsilesional primary motor cortex (M1). Therefore, iTBS was selected in this study to comprehensively elucidate its efficacy for the treatment of UL dysfunction in patients with chronic stroke.

The results of sham-controlled studies on the effect of iTBS on UL motor function in patients with chronic stroke have been controversial [13–16]. Three studies have shown that iTBS resulted in significant motor improvements [13, 15, 16], while one study revealed no difference between the iTBS and sham groups [14]. One recent meta-analysis concluded that iTBS is more effective than cTBS for stroke-related UL motor deficits [20]. However, both patients with subacute and chronic stroke were included, and Egger’s test revealed a significant publication bias [20]. To our knowledge, no previous studies have used spasticity as an outcome measure to evaluate the efficacy of iTBS combined with UL training. Furthermore, none have separately evaluated fine motor and gross motor functions of ULs. Therefore, as well as the common assessments of UL function, we also explored the effect of iTBS on these outcomes.

Motor recovery, especially fine motor, is usually limited beyond 6 months after stroke [1]. We tried to elucidate whether iTBS enhances fine motor function recovery in patients with chronic stroke. Based on previous randomized controlled trials (RCTs) of neurorehabilitation, we implemented a 2-week intervention [21, 22]. Outcome measures included the assessments of body function and activities based on the International Classification of Functioning, Disability, and Health (ICF) framework; the ICF can be a reference to determine and quantify the notion incorporated in outcome measures in stroke trials [23]. Body function was assessed using the modified Ashworth scale (MAS) [24] and Fugl-Meyer Assessment Upper Extremity (FMA-UE) [25], and activity measures used were the Action Research Arm Test (ARAT) [26], Box and Block test (BBT) [27], and motor activity log (MAL) [28]. The ARAT assesses both proximal and distal motor function and can, therefore, be used to examine fine and gross motor function separately. We hypothesized that 2-week iTBS combined with standard rehabilitation programs would induce greater improvements in the FMA and in spasticity in patients with chronic stroke compared to a sham intervention. We also expected that iTBS would result in greater improvements in the ARAT, BBT, and MAL. To our knowledge, this is the first RCT that compared the functional improvements in body function and activity domains, including spasticity, fine/gross motor function, and activities of daily living (ADL), post-stroke in patients undergoing real vs. sham iTBS.

## Methods

### Participants

Patients with stroke were enrolled from a rehabilitation department between 2013 and 2016. The diagnosis was confirmed by the authors according to clinical manifestations and imaging studies. Inclusion criteria for patients were as follows: (1) aged between 30 and 70 years; (2) first-ever ischemic or haemorrhagic stroke; (3) onset ≥6 months; (4) unilateral cerebral stroke with hemiplegia or hemiparesis. Exclusion criteria were as follows: (1) brainstem or cerebellar stroke; (2) active psychiatric diseases; (3) severe psychological impairments, such as mental retardation, autism, or severe communication problems; (4) cognitive impairments that may interfere with understanding instructions; (5) progressive disorders, such as neurodegenerative disease; (6) an active medical condition, such as infection; (7) a history of seizure or aneurysm; (8) metal head implants.

All patients provided written informed consent. The study protocol was performed in accordance with the Declaration of Helsinki and was approved by a local institutional review board. This trial is registered under ClinicalTrials.gov ID No. NCT01947413.

### Design and procedures

**Fig. 1:**
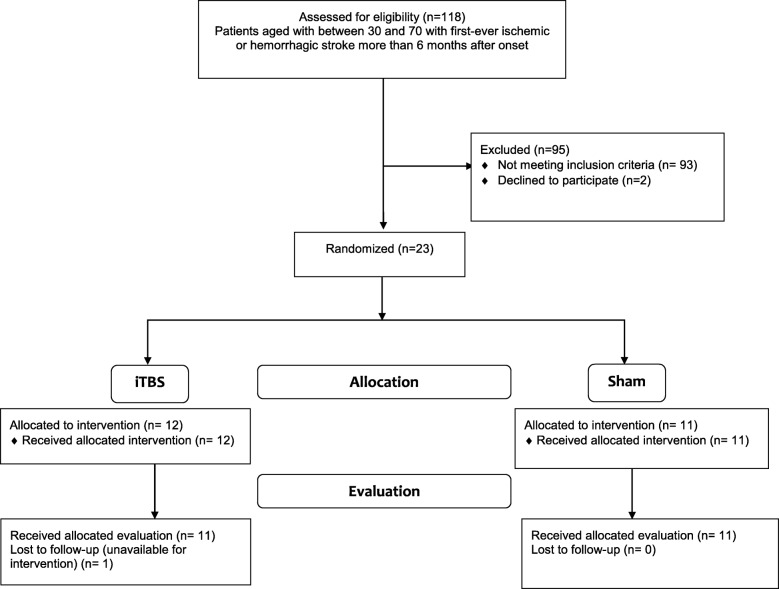
Consort diagram of participant recruitment and randomization

**Fig. 2:**
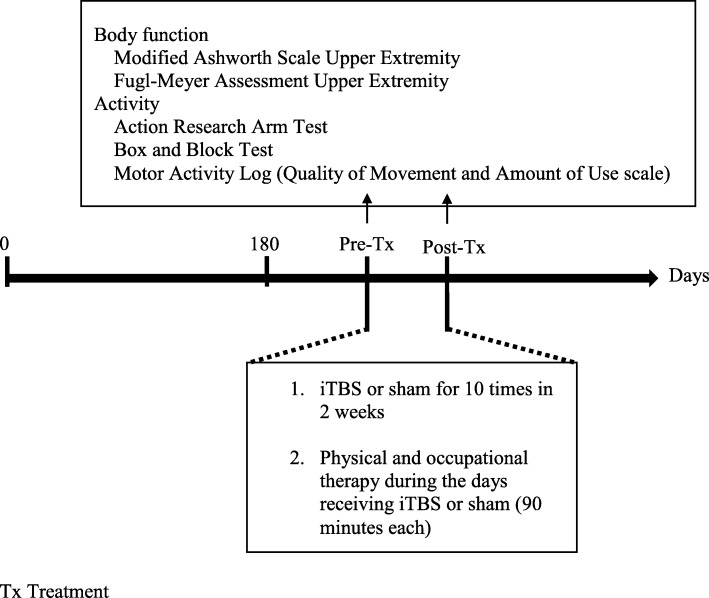
Study design

This study was a prospective, randomized, sham-controlled trial. Patients were blind to whether they received iTBS or sham-iTBS five times/week for 2 consecutive weeks. The flow diagram of the randomization procedure and the study design are illustrated in Figs. 1 and 2, respectively. The raters (occupational therapists who were only in contact with the patients during assessment), blinded to group assignment, were trained to administer outcome measures by senior therapists prior to the experiment, and passed a written competency and reliability test. Ten patients were selected for the intra-rater and inter-rater reliability tests at a 7-day interval. The intraclass correlations for intra-rater/inter-rater reliability of the MAS-UE, FMA-UE, ARAT, and BBT were 0.841/0.841, 0.984/0.992, 0.986/0.998, and 1.000/0.998, respectively.

The outcome measures were administered within 3 days before and after completing the 2-week iTBS/sham iTBS. All patients continued to participate in the standard in-patient rehabilitation programs, including conventional physical and occupational therapy (90 min/session, 5 sessions/week), on the same day as the interventions. Demographic and clinical characteristics, such as age, sex, affected limb, stroke type, and lesion side, were recorded (Table 1).

**Table 1:** Demographic and clinical data

Variables		iTBS (*n* = 11)	Control (*n* = 11)	*p* value
Age (years)		52.9 ± 11.1	52.6 ± 8.3	0.937^a^
Gender		7/4	7/4	> 0.99^b^
Clinical characteristics				
Stroke type				
Hemorrhage		9 (82%)	8 (73%)	> 0.99^b^
Infarction		2 (18%)	3 (27%)	
Stroke side				
Right		5 (45%)	2 (18%)	0.170^b^
Left		6 (55%)	9 (82%)	
Stroke location				
Supratentorial		4 (36%)	3 (27%)	0.647^b^
Infratentorial		7 (64%)	8 (73%)	
MMSE		26.9 ± 3.4	23.8 ± 7.3	0.220^a^
NIHSS		3.7 ± 2.5	5.9 ± 5.6	0.251^a^
Medications				
Antispasticity		2 (18%)	3 (27%)	0.611^b^

Data are expressed as mean ± SD or N (%)

^a^*t* tests;^b^ *Chi-square* tests

iTBS intermittent theta burst stimulation, MMSE Mini-mental state examination, NIHSS National Institutes of Health Stroke Scale

### iTBS protocol

iTBS was applied to the hand motor area of the affected hemisphere using a handheld figure-of-eight coil (70-mm standard coil; Magstim, Whitland, Dyfed, UK) connected to a Magstim Rapid^2^ stimulator. The optimal location of the coil was determined by the location on the scalp where the magnetic stimulation produced the largest MEP from the contralateral first dorsal interosseous muscle. True stimulation at 80% active motor threshold (AMT) targeted the identified hotspot, with the coil held tangentially to the skull, orientated at 45° to the midsagittal axis, inducing posterior-anterior current in the brain. AMT was assessed before each intervention and was defined as the lowest transcranial magnetic stimulation (TMS) intensity to elicit MEPs of a 200 μV peak-to-peak amplitude in 5 of 10 successive trials during slight contraction (10–20% of maximum) of the target muscle. Sham stimulation was administered on the same site with the coil flipped over at a lower intensity (60% AMT), which results in a lower output equivalent to that of the normal site at approximately 46.8% AMT, because the output of the flip side is about 78% of the normal side [29]. Previous study demonstrated that a single theta burst with intensity lower than 70% AMT has no effect on MEPs [30]. The sound and sensation were similar to real stimulation so that the patients cannot differentiate. Similar sham stimulation method has been applied in several previous studies and proved to be useful [29, 31]. When MEPs could not be measured from the affected side, iTBS was applied to the mirror location of the hotspot of the sound hemisphere (non-lesional M1) at the highest intensity that our Rapid^2^ could generate for TBS (51% of maximum stimulator output (MSO)). Patients who had no measurable MEP on the affected side and who required stimulus intensity (80% AMT) higher than 51% of MSO were also stimulated at 51% of MSO. No patient was able to distinguish whether they had received real or sham stimulation. Patients were instructed to relax their hands at all times during the experiment once the AMT had been recorded.

Real or sham iTBS was delivered at the same time of day for 5 consecutive days per week for 2 weeks. iTBS gave a 2-s train of bursts, which contained three 50-Hz pulses repeated every 200 ms (i.e., 5 Hz) at an intensity of 80% AMT, every 10 s for 20 times (600 pulses in total) [8]. Similar stimulation parameters have been applied in several previous studies [21, 32].

### Outcome measures

#### MAS

MAS [24] was used for determining muscle spasticity. We summed the MAS scores of the affected elbow, wrist, and finger flexor muscles to evaluate UL spasticity. 1+ was calculated as 1.5 for summation.

#### FMA-UE

The FMA [25] is a performance-based quantitative evaluation specifically used for measuring post-stroke impairments, including motor function, balance, sensation, and joint functions, and UL motor function evaluation was selected in the current study.

#### ARAT

The ARAT [26] is a measure of UL motor function after stroke which comprises of 4 subsections: grasp, grip, pinch, and gross movement.

#### BBT

The BBT [27] assesses grasping, transporting, and releasing of small objects to determine unilateral gross manual dexterity.

#### MAL

The MAL [28] is a structured interview that measures the use of the impaired arm in patients with stroke, which is used to obtain information about 14 activities of daily living.

### Statistical analysis

Data analyses were conducted by H.L. Peng who was blinded to group allocation. To determine the baseline comparability of demographic characteristics, chi-square tests and independent two-sample t-tests were applied to categorical and continuous variables, respectively. Analysis of covariance was used to test whether the iTBS group showed greater improvement than the control group after treatment. We defined pre-treatment performance as the covariate, group as the independent variable, and post-treatment performance as the dependent variable. Effect size (*η*^*2*^) was calculated for each outcome variable to indicate the degree of group differences. A large effect is represented by an *η*^*2*^ of at least 0.138, a moderate effect by an *η*^*2*^ of 0.059, and a small effect by an *η*^*2*^ of 0.01 [33]. Significance was set at 0.05 (one-tailed).

## Results

A total of 118 patients were screened. Twenty-three were eligible for inclusion and were randomly assigned to the iTBS or control group by a research assistant using a computer random number generator. Of these 23, one patient in the iTBS group was lost to follow-up due to unavailability for intervention. Finally, 11 patients in the iTBS group and 11 in the sham-iTBS group completed the intervention and evaluation. There were no adverse events throughout the study course. There was no significant between-group difference before treatment on demographic characteristics (Table 1) or outcome measures (Table 2). AMT cannot be measured in 4 patients in the iTBS group and 5 patients in the control group. The mean ± standard deviation from the obtained AMT data of iTBS and control groups were 67% ± 11 and 71% ± 8%, respectively.

**Table 2:** Descriptive and inferential statistics for analysis of outcome measures

Variables	Pre-treatment			Post-treatment		ANCOVA		
	iTBS (*n* = 11)	Control (*n* = 11)	*p* value (*t* test)	iTBS (*n* = 11)	Control (*n* = 11)	*F1,19*	*p* value	Effect size *η2*
MAS-UE	3.90 ± 2.10	4.05 ± 1.56	0.861	3.30 ± 1.98	4.29 ± 1.44	5.8	0.014*	0.233
FMA-UE	33.33 ± 19.80	30.03 ± 22.11	0.732	34.65 ± 19.80	27.06 ± 20.79	3.4	0.046*	0.151
ARAT	18.62 ± 19.38	16.72 ± 22.8	0.834	23.18 ± 20.71	13.3 ± 19.95	16.3	0.001†	0.460
Gross movement	9.06 ± 7.08	7.98 ± 6.48	0.708	10.02 ± 6.60	6.72 ± 6.18	2.1	0.083	0.099
Grasp	4.00 ± 4.48	3.52 ± 5.28	0.819	4.60 ± 4.96	2.68 ± 4.72	7.6	0.006†	0.286
Grip	4.80 ± 7.08	5.58 ± 7.86	0.800	6.42 ± 7.20	5.04 ± 7.38	3.7	0.035*	0.163
Pinch	2.46 ± 3.39	1.86 ± 3.51	0.692	3.51 ± 3.21	1.26 ± 2.82	11.9	0.002†	0.384
BBT	10.20 ± 16.80	9.00 ± 21.00	0.920	11.40 ± 18.60	9.60 ± 22.20	3.7	0.036*	0.161
MAL (AOU)	10.44 ± 16.38	18.9 ± 22.96	0.460	12.18 ± 14.70	20.72 ± 22.54	0.02	0.460	0.001
MAL (QOM)	10.50 ± 13.44	15.26 ± 22.40	0.580	10.36 ± 13.86	21.28 ± 22.12	0.5	0.246	0.031

Values are expressed as mean ± SD

iTBS intermittent theta burst stimulation, MAS-UE Modified Ashworth Scale Upper Extremity, FMA-UE Fugl-Meyer Assessment Upper Extremity, ARAT Action Research Arm Test, BBT Box and Block test, MAL(AOU) Motor Activity Log (Amount of Use), MAL (QOM) Motor Activity Log (Quality of Movement)

*p < 0.05; †*p* < 0.01

**Fig. 3:**
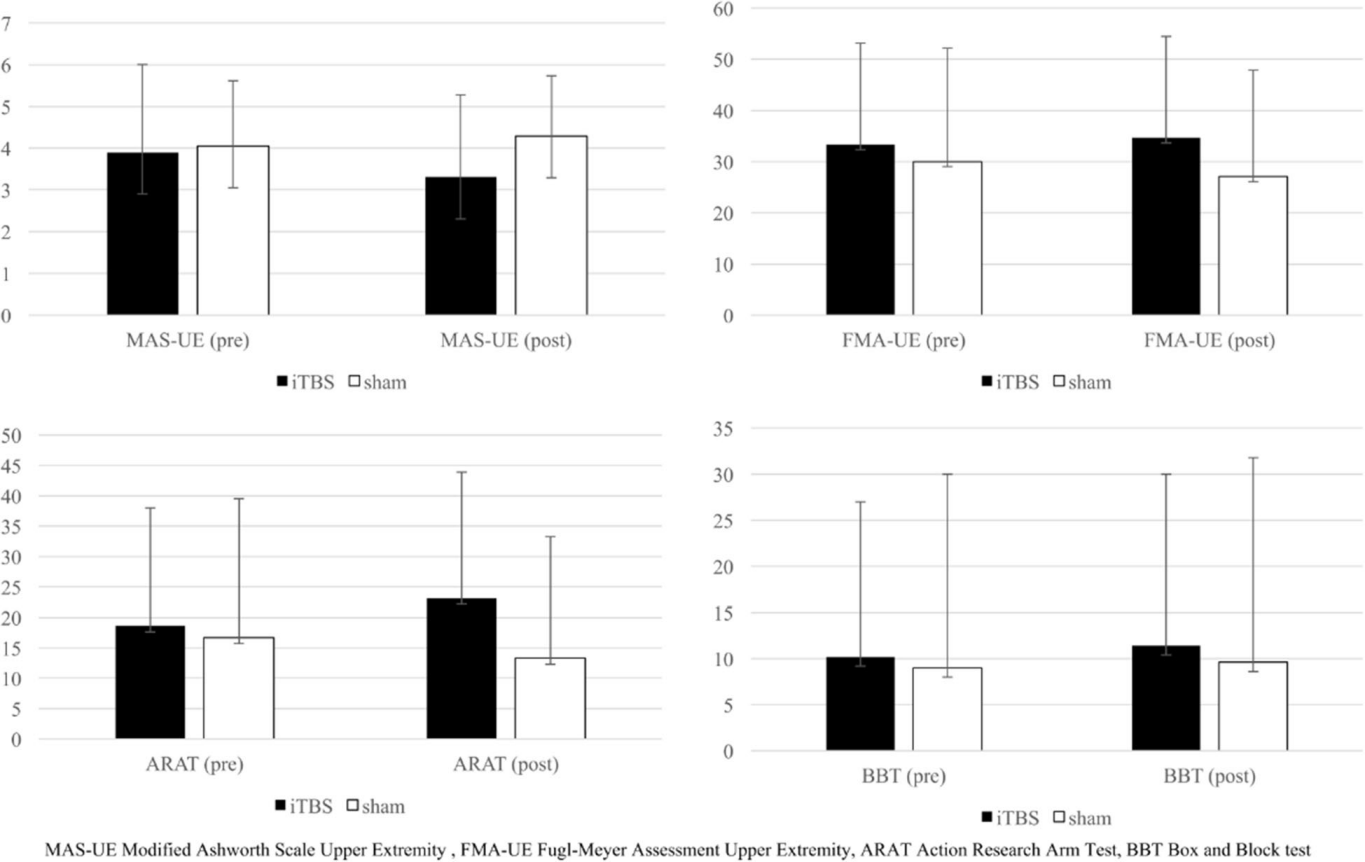
Changes in upper limb motor function before and after intervention

The iTBS group had greater gains in the MAS and FMA, with larger effects than the control group after the intervention (MAS: F_1,19_ = 5.8, *p* = 0.014, *η*^*2*^_=_0.233; FMA-UE: F_1,19=_3.4, *p* = 0.046, *η*^*2*^ = 0.151) (Table 2, Fig. 3). Moreover, the iTBS group had greater gains in the ARAT in various domains, with larger effect sizes than the control group (average: F_1,19_ = 16.3, *p* = 0.001, *η*^*2*^ = 0.460; grasp: F_1,19_ = 7.6, *p* = 0.006, *η*^*2*^ = 0.286; grip: F_1,19_ = 3.7, *p* = 0.035, *η*^*2*^ = 0.163; pinch: F_1,19_ = 11.9, *p* = 0.002, *η*^*2*^ = 0.384).

After treatment, the iTBS group had significantly greater gains in the BBT with a large effect size than the control group (F_1,19_ = 3.7, *p* = 0.036, *η*^*2*^_=_0.161), although the BBT score of both groups improved. There were no significant between-group differences in the changes in the MAL Amount of Use (AOU) scale and MAL Quality of Movement (QOM) scale.

## Discussion

In this pilot study, iTBS induced greater gains in the MAS and FMA than sham stimulation, and more greatly improved BBT and ARAT scores, especially for fine motor function. However, the changes in the MAL did not differ between the two groups. These findings suggest that iTBS combined with conventional neurorehabilitation reduces spasticity and improves UL motor function, but not ADL, in patients with chronic stroke. The beneficial effect of iTBS in patients with chronic stroke is even more striking if we consider that the likelihood of fine motor recovery is lower in chronic phases than in the subacute or acute stroke. Overall, although limited by the relatively small sample size, these findings suggest that iTBS may be used as an adjunct therapy to enhance UL function in patients with chronic stroke.

### The effect of iTBS on upper limb motor function

The application of iTBS to the ipsilesional hemisphere enhanced UL motor function, as measured by the FMA, ARAT, and BBT. The iTBS-induced changes in these outcome measures achieved large effect sizes, although the changes in FMA and ARAT were not greater than the minimal clinically important difference [34, 35]. These results are consistent with those of previous studies on iTBS [13–15] and conventional high-frequency facilitatory rTMS [22, 36] in stroke patients, although iTBS may not enhance UL motor function in patients with incomplete spinal cord injury [37]. Our results may be explained by vicariation [38, 39]. The vicariation theory postulates that brain areas are reorganized to substitute the functions of nearby injured areas [38, 39]. Considering this compensatory neural plasticity, facilitatory stimulation should be applied to the affected hemisphere in order to enhance excitability of compensatory neurons [39]. According to this model, facilitation of the ipsilesional hemisphere may stimulate cerebral tissue reorganization and restore the balance of motor cortical excitability between hemispheres, thus leading to functional recovery of the affected limb.

Our findings that the iTBS group had greater improvements than the control group, in pinch, grasp, and grip functions, but not in gross movement, suggest that iTBS can enhance fine UL movement to a greater degree than gross UL movement. Our results were partially compatible with those of previous iTBS studies [13–16]. For example, Talelli et al. first applied iTBS as an intervention to improve UL motor function recovery in 6 patients with chronic stroke, and found that simple reaction times of gripping a dynamometer were improved [13]. Ackerley et al. recruited 10 patients with subcortical CS and found that iTBS followed by motor training enhanced performance in grip-lift kinetics of the affected hand [15]. Another study by Ackerley et al. also revealed that iTBS induced significant improvements in the ARAT [16]. However, another study by Talelli et al. demonstrated that there were no significant differences in the grasp and pinch strength or performance in the Nine-Hole Peg Test and Jebson Taylor Test between iTBS and sham groups post-treatment and at all follow-ups (4, 30, and 90 days), although motor performance slightly improved in both groups [14]. It is generally accepted that the proximal limb motor (gross motor) function recovers better than the function of distal parts (fine motor) after stroke [40], possibly because the recovery of gross motor function can be mitigated by multiple neural pathways [41], while fine motor function is primarily controlled locally by the M1 and corticospinal tract [42]. We selected the first dorsal interosseous muscle for AMT determination, which mainly accounts for fine motor function, and this may account for our results. However, gross motor movement is dependent on shoulder and elbow function. Our findings suggest that iTBS enhances fine motor recovery, even in patients with chronic stroke.

This is the first study to verify the efficacy of iTBS combined with UL training in reducing spasticity in patients with chronic stroke. It has been postulated that impaired cortical motor neurons after stroke cannot sustain inhibitory signals to the corticospinal tract, leading to an increased spinal motor neuron excitability that results in spasticity [43]. Facilitatory rTMS, including iTBS, has been reported to reduce spasticity in patients with multiple sclerosis [44, 45], cerebral palsy [46], and spinal cord injury [47, 48]. In patients with acute and chronic stroke, Kim et al. found that a single session of iTBS resulted in a transient reduction of spasticity [49]. In another study with patients with chronic stroke, facilitatory rTMS decreased the F-waves, implying that facilitatory rTMS suppresses the excitability of the spinal cord by enhancing inhibitory inputs from the cortex to spinal neurons [50]. In addition, iTBS may cause changes in endogenous neurotransmitters including γ-aminobutyric acid [51], glutamate [52], and dopamine [53], which are involved in synaptic plasticity, thereby increasing the efficacy of synaptic transmission [54]. As a result, it may facilitate inhibitory interneurons from the cortex to the spinal cord and reduce spasticity. Further trials with additional neurophysiological studies to examine the effects induced by iTBS on corticospinal tract excitability are warranted to uncover its underlying neuronal mechanisms and long-term clinical effects.

The iTBS group had greater gains in the BBT than the control group, which indicates that iTBS may enhance the recovery of gross manual dexterity of UL in patients with chronic stroke. Our results were not compatible with those of a previous iTBS study [14], but partially compatible with those of rTMS studies [55, 56]. Namely, Talelli et al. revealed no difference in the Nine Hole Peg Test after intervention between the iTBS and sham groups in patients with chronic stroke [14]. One meta-analysis showed no significant differences in the BBT between rTMS and conventional rehabilitation programs [7]. However, one RCT found that the facilitatory rTMS group had significantly greater improvements on BBT than a sham group, up until 6 months post-intervention [55]. Overall, these variable findings may be due to different patient characteristics, stimulation protocols, and/or stimulation locations among studies. Furthermore, the effects of rTMS on neural activity depend on factors such as coil orientation, stimulation intensity, and brain activation [57]. For example, the TMS-related effect has been linked to the total number of stimuli, whereby a profound change in cortical excitability was observed for longer periods of rTMS [58]. TBS and paired associative stimulation modulate different synapses in the motor cortex, although both modulate cortical excitability [59]. Our finding suggests that iTBS may be used to enhance the gross manual dexterity in patients with chronic stroke.

In the current study, MAS, FMA, and ARAT scores was decreased after intervention in the control group, which were also demonstrated in previous literature using a different sham stimulation method [16]. One possible explanation is that the facilitatory effect of iTBS outweigh the exhaustibility from rehabilitation, so the exhaustibility caused by extensive rehabilitation is only seen in the control group having no improvement.

The iTBS group did not show greater improvement in the MAL (QOM) or MAL (AOU) than the control group. This is consistent with previous facilitatory rTMS results [60]. Malcolm et al. failed to identify a superiority of 10 sessions of 20 Hz rTMS to the ipsilesional hemisphere over a sham intervention in terms of MAL (QOM) and MAL (AOU) at 2-week and 6-month follow-ups [55]. MAL has been used to capture changes in the actual use of the affected arm that differ from compensatory strategies post stroke, and is dissimilar to laboratory-based function measurements [61]. A possible explanation for this result is that the follow-up duration was too short to detect the ADL changes and learned non-use, thus reinforcing the use of compensatory strategies despite gradual functional recovery [62].

### Study limitations

There were several limitations to this study. First, as with other iTBS studies [13–15, 21], the sample size was relatively small; the results should be confirmed in a larger-scale study. Second, the follow up duration was too short to assess the long-term impact of iTBS; future studies should also investigate the persistence of these effects. Third, real or sham iTBS was arranged at the available time based on the daily regime of neurorehabilitation. It is not clear whether the effect of iTBS is associated with the stimulation timing relative to neurorehabilitation or not. Fourth, high AMT was likely due to the severely damaged motor pathway. Such high motor thresholds led to insufficient stimulus intensity due to the limitation of the TMS machine, and could lower the efficacy of iTBS. Fifth, the sham stimulation method allows the intensity to be delivered at a decreased magnitude rather than abolishing it. In addition, the sensation on the scalp may be similar but could not be identical with that of the iTBS group.

## Conclusions

iTBS over the affected hemisphere reduced spasticity and improved UL motor function, particularly fine motor function, which is not usually the case in the chronic stage of stroke. To conclude, iTBS is a non-invasive and safe neuromodulatory stimulation technique that may be a beneficial adjunct to neurorehabilitation for patients with chronic stroke who have relatively low potential for motor recovery. While the current study is a pilot study with small sample size, further larger-scale study is needed to confirm the findings.

## Abbreviations

ADL: Activities of daily living
AMT: Active motor threshold
AOU: Amount of Use scale
ARAT: Action Research Arm Test
BBT: Box and Block test
cTBS: Continuous TBS
FMA-UE: Fugl-Meyer Assessment Upper Extremity
ICF: International Classification of Functioning, Disability, and Health framework
iTBS: Intermittent TBS
M1: Primary motor cortex
MAL: Motor activity log
MAS: Modified Ashworth scale
MEPs: Motor-evoked potentials
QOM: Quality of Movement scale
RCT: Randomized controlled trial
rTMS: Repetitive transcranial magnetic stimulation
TBS: Theta burst stimulation
TMS: Transcranial magnetic stimulation
UL: Upper limb
